# First report of an intersex individual of the click beetle *Pectocerafortunei* (Elateridae) and additional cases of gynandromorphism in Coleoptera (Lucanidae, Scarabaeidae)

**DOI:** 10.3897/BDJ.13.e144929

**Published:** 2025-02-20

**Authors:** Donguk Kim, Sangeun Hyun, Kwang Shik Choi

**Affiliations:** 1 Department of Biology, College of Natural Sciences, Kyungpook National University, Daegu, Republic of Korea Department of Biology, College of Natural Sciences, Kyungpook National University Daegu Republic of Korea; 2 School of Life Sciences, BK21 FOUR KNU Creative BioResearch Group, Kyungpook National University, Daegu, Republic of Korea School of Life Sciences, BK21 FOUR KNU Creative BioResearch Group, Kyungpook National University Daegu Republic of Korea

**Keywords:** sexual dimorphism, stag beetle, rhinoceros beetle, *Dorcus*, *Trypoxylus*, Korea

## Abstract

**Background:**

The occurrence of individuals exhibiting both male and female phenotypes is a rare phenomenon observed across various insect orders.

**New information:**

This study documents the first case of an intersex individual of *Pectocerafortunei* Candèze, 1873, from the Korean Peninsula, including behavioural findings. This is the first report of such a phenomenon in the family Elateridae. Two cases of gynandromorphism are also described: one in *Dorcustitanuscastanicolor* (Motschulsky, 1861) (Lucanidae) and the other in *Trypoxylusdichotomusseptentrionalis* (Kôno, 1931) (Scarabaeidae). The former is a new record for this subspecies. An updated list of intersex and gynandromorphic beetles is also provided.

## Introduction

In sexually dimorphic species, a rare phenomenon has been observed in which certain arthropod individuals exhibit both male and female phenotypic characteristics. Although this phenomenon is uncommon, it has been documented across most insect orders (Narita et al. 2010, Lightburn et al. 2022). Such individuals can be classified into two types: gynandromorphs, in which distinct boundaries separate male and female parts and intersexes, in which such boundaries are ambiguous or absent (Narita et al. 2010, Fusco and Minelli 2023). These individuals exhibit diverse and unique morphological characteristics, with male and female phenotypes clearly separated bilaterally or transversally or mixed in a mosaic pattern (Fusco and Minelli 2023).

The phenomena of intersex and gynandromorphism have various causes and can be classified according to different criteria depending on the characteristics of each case or the researcher’s approach. Sex determination and differentiation result from the complex interplay of genetic, developmental and environmental factors. These phenomena may be induced by distal factors, such as hybridisation or parasitic infection or proximal factors, such as the missing expression of a key sex-determining gene (Fusco and Minelli 2023).

In the order Coleoptera, several cases of intersex and gynandromorphic individuals have been reported, with most records belonging to the family Scarabaeidae (Narita et al. 2010). However, since the first documented case of gynandromorphism in Coleoptera by Wickham (1903), no such cases have been identified in click beetles. In this study, we report the first case of an intersex individual of *Pectocerafortunei* Candèze, 1873, discovered in the Korean Peninsula, marking the first record of such a case within the family Elateridae. Moreover, we report two cases of gynandromorphism: one in *Dorcustitanuscastanicolor* (Motschulsky, 1861) (Lucanidae) and the other in *Trypoxylusdichotomusseptentrionalis* (Kôno, 1931) (Scarabaeidae). The former case represents a new record for this subspecies. To provide an updated checklist of intersex and gynandromorphic cases in Coleoptera, we combined previous reports with our findings, resulting in the recognition of 31 species across eight families.

## Materials and methods

**Figure 1.**
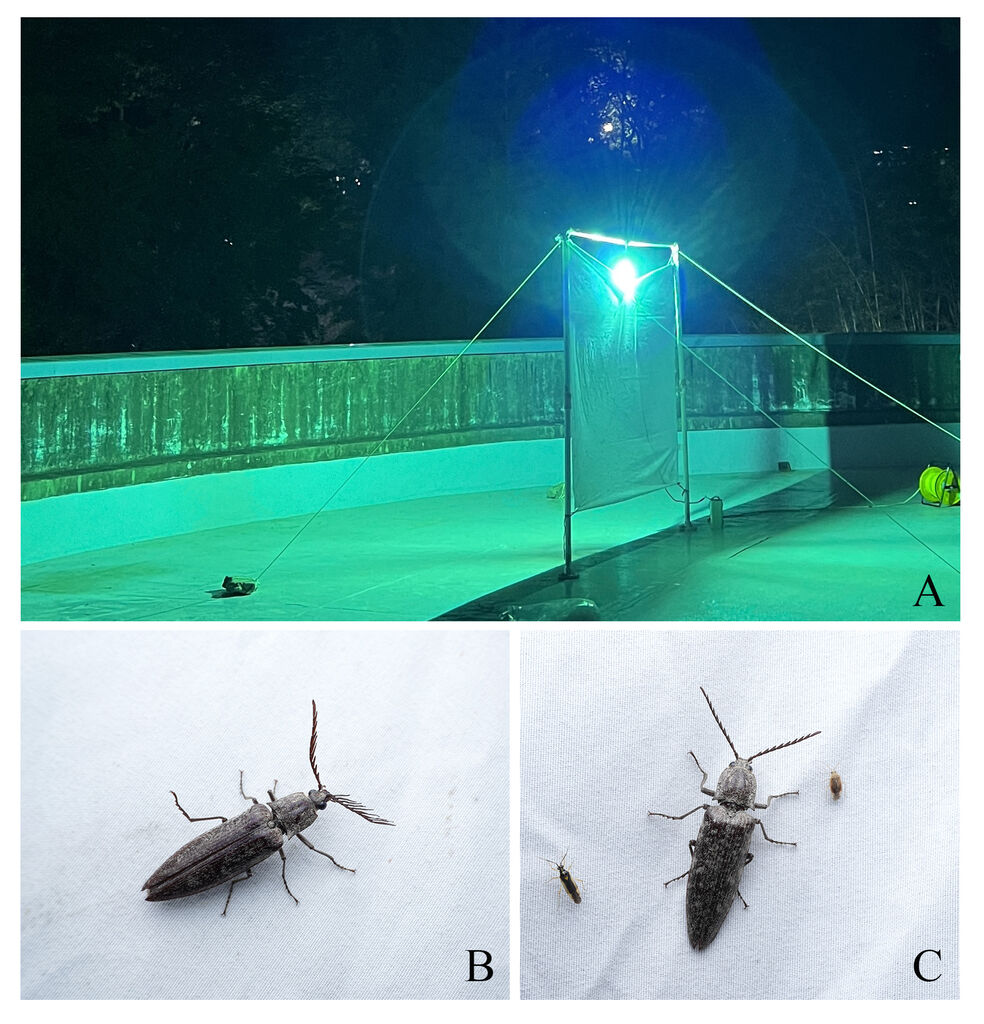
Field photograph of *Pectocerafortunei* at CNU. **A** Light trap installation setup; **B** Intersex individual of *Pectocerafortunei* attracted to the light trap; **C** Normal female individual of *Pectocerafortunei* attracted to the light trap.

An intersex individual of *P.fortunei* was collected at Chungnam National University (CNU, Daejeon, Korea), through a light trap survey in May 2023 (Fig. 1). Two gynandromorphs (*D.titanuscastanicolor* and *T.dichotomusseptentrionalis*) were found in a private insect breeding facility in July 2019 and November 2019, respectively.

The genitalia of all specimens were dissected using forceps and the surrounding tissues were removed by treating them with a 10% potassium hydroxide (KOH) solution for 50 min. The specimens were preserved as dry specimens and the genitalia were stored in microtubes containing glycerine for long-term preservation.

External morphological characteristics and genitalia were examined and photographed using an Olympus SZX16 stereomicroscope (Olympus, Tokyo, Japan), an Olympus OMD EM10 Mark II digital camera and a Michrome 16 CMOS camera (Tucsen, Fujian, China). Adobe Photoshop 21.2.0 (Adobe Systems Inc.) was used to edit the captured images. The specimens are deposited in the Laboratory of Animal Systematics and Taxonomy, School of Life Sciences, College of Natural Sciences, Kyungpook National University (KNU, Daegu, Korea).

The behavioural observation of *P.fortunei* was conducted by introducing an intersex individual and a typical female individual into a single breeding case lined with tissue paper (Suppl. material 1).

The updated list of intersex and gynandromorphic cases in Coleoptera was compiled by reviewing the literature provided by Narita et al. (2010), incorporating our findings, previously missing studies and recent literature.

## Taxon treatments

### Pectocera fortunei

Candèze, 1873

BFF4CEDF-2AB8-588E-B428-FA17CDFFF02A

#### Materials

- **Type status:** Other material. **Occurrence:** sex: 1 intersex; occurrenceID: 52C9BE44-7BEA-5418-BC0A-7598D646DA89; **Taxon:** genus: Pectocera; specificEpithet: *fortunei*; scientificNameAuthorship: Candèze, 1873; **Location:** country: South Korea; locality: CNU, Gung-dong, Yuseong-gu, Daejeon, Sang Eun Hyun leg.; **Event:** eventDate: 25.V.2023; **Record Level:** basisOfRecord: Preserved Specimen
- **Type status:** Other material. **Occurrence:** sex: 5 females; occurrenceID: DCF7A291-7B77-54A4-A0F3-BF14635E96E8; **Taxon:** genus: Pectocera; specificEpithet: *fortunei*; scientificNameAuthorship: Candèze, 1873; **Location:** country: South Korea; locality: CNU, Gung-dong, Yuseong-gu, Daejeon, Sang Eun Hyun leg.; **Record Level:** basisOfRecord: Preserved Specimen
- **Type status:** Other material. **Occurrence:** sex: 1 male, 1 female; occurrenceID: EE66458A-FC82-55DB-AAB4-CB64DBE9B5E7; **Taxon:** genus: Pectocera; specificEpithet: *fortunei*; scientificNameAuthorship: Candèze, 1873; **Location:** country: Japan; locality: Aseri, Kyotanba Town, Kyoto Pref., 14.VI.2014, Satoshi Kubo leg.; **Record Level:** basisOfRecord: Preserved Specimen

#### Description

##### Morphology of the intersex *Pectocerafortunei*

**Figure 2.**
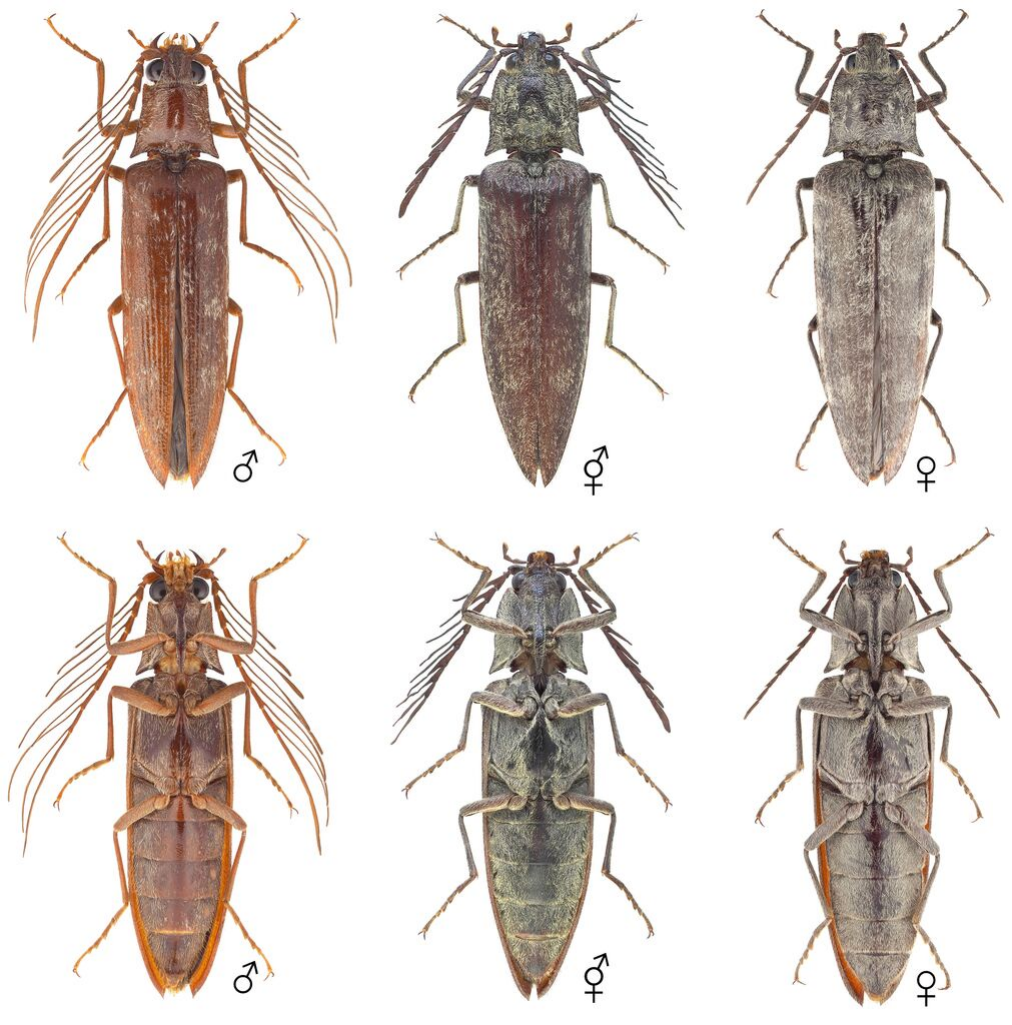
Habitus of *Pectocerafortunei*, dorsal (above) and ventral (below) views. From left: male, intersex and female.

**Figure 3.**
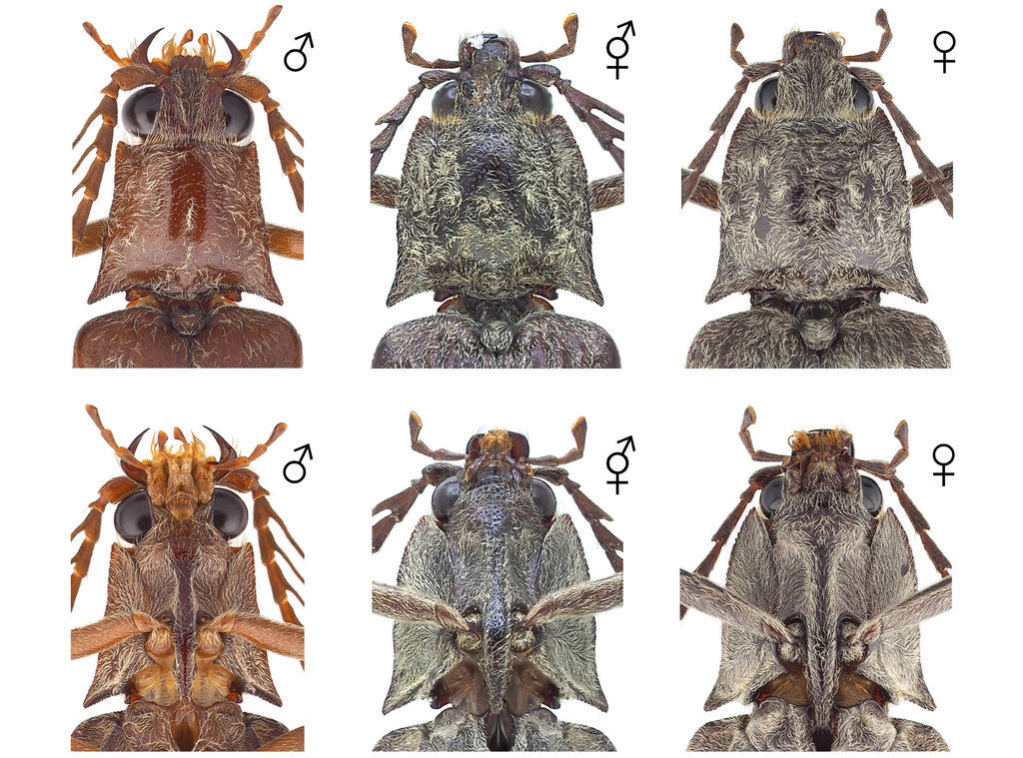
Pronotum and head of *Pectocerafortunei*, dorsal (above) and ventral (below) views. From left: male, intersex and female.

**Figure 4.**
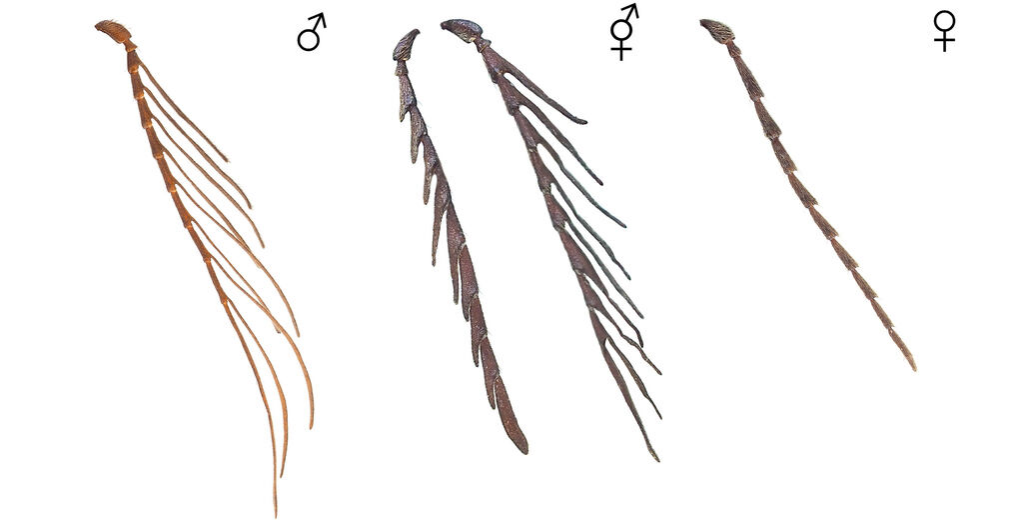
Antennae of *Pectocerafortunei*. From left: male, intersex and female.

**Figure 5.**
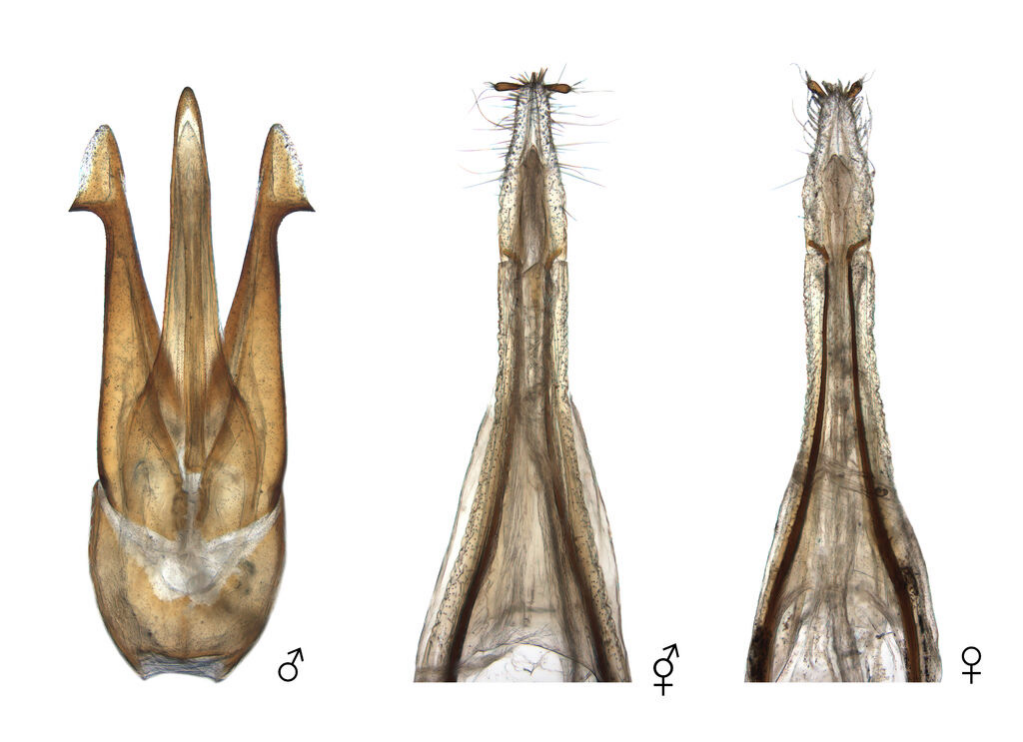
Genitalia of *Pectocerafortunei*. From left: male, intersex and female.

The body overall exhibits female traits, except for the antennae (Figs 2, 3, 4). The head displays relatively small eyes and mandibles resembling those of a typical female (Fig. 3). The lateral margins of the pronotum are convex and arcuate, narrowing towards the posterior angles, as typically seen in females (Fig. 3). The antennae are pectinate, similar to those of a typical male and asymmetrical between the left and right sides, with nine irregularly transformed flagellomeres (Fig. 4). The genitalia are fully developed and identical to a typical ovipositor (Fig. 5).

##### Behavioral interactions between an intersex and a typical female

To examine the behavioural interactions between an intersex individual and a typical female, one of each was placed in a single breeding case for observation. As a result, they exhibited only biting and aggressive behaviour, with no mating behaviour observed.

#### Notes

In *P.fortunei*, the prominent male sexual characteristics include pectinate antennae, relatively large eyes and a narrow pronotum (Figs 2, 3). In the observed specimen, only female sexual characteristics were displayed in all body parts, including the genitalia, except for the antennae (Figs 2, 3, 4, 5). The antennae exhibited a pectinate pattern similar to that of a male. However, the nine flagellomeres appeared as if the female flagellomeres had been transformed into a slender and elongated shape like those of a male (Fig. 4). Consequently, it was ambiguous to clearly identify this as a distinct male trait; thus, the specimen was classified as intersex. This is the first record for this family.

### Dorcus titanus castanicolor

B4E31182-14C5-5D7C-BE81-688889D50C43

#### Materials

- **Type status:** Other material. **Occurrence:** sex: 1 gynandromorph; occurrenceID: 09268E7D-25D3-5F13-A399-753809D0B384; **Taxon:** genus: Dorcus; specificEpithet: *titanuscastanicolor*; scientificNameAuthorship: (Motschulsky, 1861); **Location:** country: South Korea; locality: Hyeongsu Kim leg.; **Event:** eventDate: VII.2019 (breed); **Record Level:** basisOfRecord: Preserved Specimen
- **Type status:** Other material. **Occurrence:** sex: 1 female; occurrenceID: 9E55012A-86F6-5022-A53D-93EF9D0DF949; **Taxon:** genus: Dorcus; specificEpithet: *titanuscastanicolor*; scientificNameAuthorship: (Motschulsky, 1861); **Location:** country: South Korea; stateProvince: Chungcheongbuk-do; locality: Chupungryeong, Chupungryeong-myeon, Yeongdong-gun, Seung Mo Lee Coll.; **Event:** eventDate: 29.VII.1972; **Record Level:** basisOfRecord: Preserved Specimen
- **Type status:** Other material. **Occurrence:** sex: 1 male, 1 female; occurrenceID: 95F2493E-FB09-5626-8E45-ECAC995F39D8; **Taxon:** genus: Dorcus; specificEpithet: titanuscastanicolor; scientificNameAuthorship: (Motschulsky, 1861); **Location:** country: South Korea; stateProvince: Jeollanam-do; locality: Is. Jindo, Jukrim-ri, Imhoe-myeon, Jindo-gun, Donguk Kim leg.; **Event:** eventDate: 4.VIII.2020; **Record Level:** basisOfRecord: Preserved Specimen

#### Description

##### Morphology of the gynandromorph *Dorcustitanuscastanicolor*

**Figure 6.**
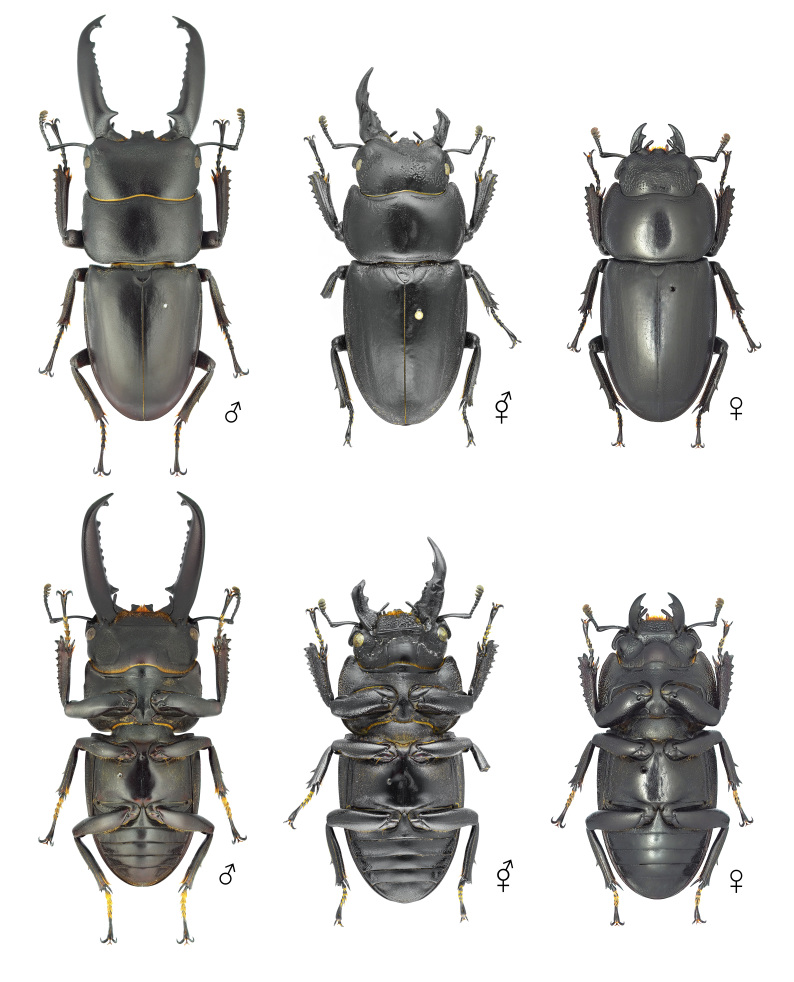
Habitus of *Dorcustitanuscastanicolor*, dorsal (above) and ventral (below) views. From left: male, gynandromorph and female.

**Figure 7.**
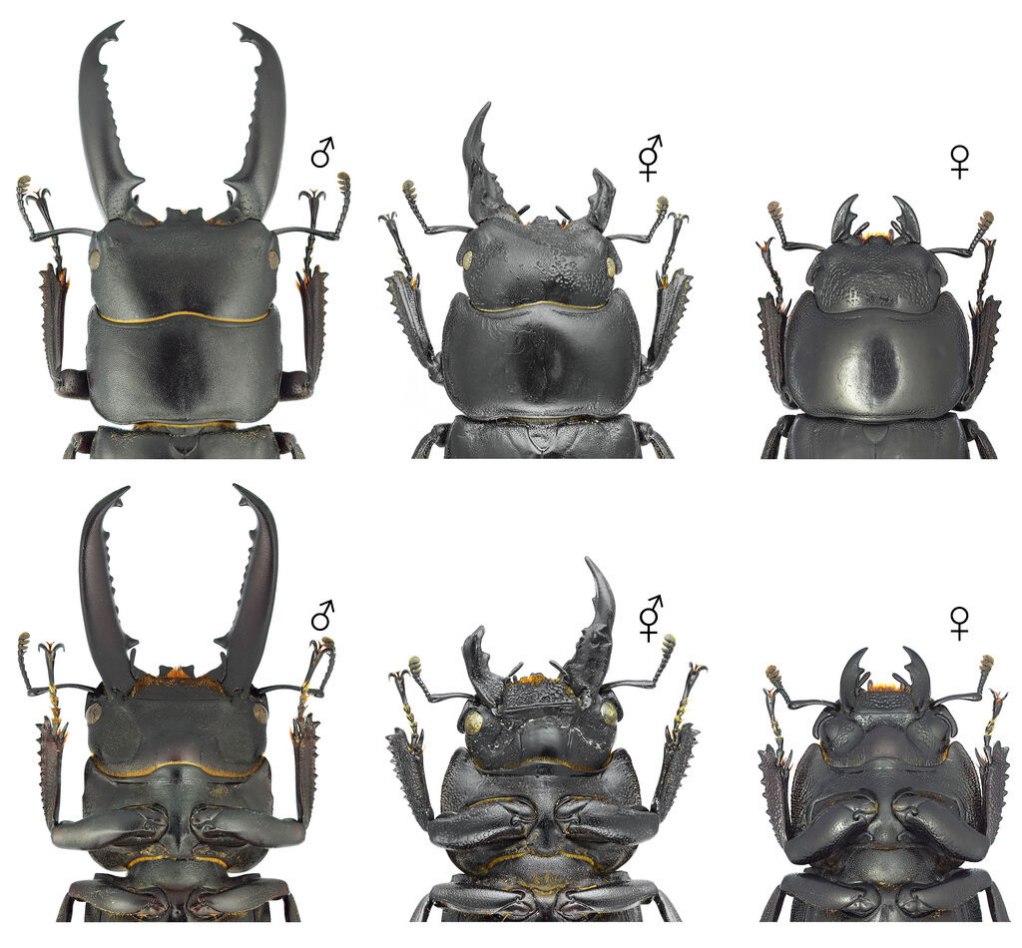
Pronotum and head of *Dorcustitanuscastanicolor*, dorsal (above) and ventral (below) views. From left: male, gynandromorph and female.

**Figure 8.**
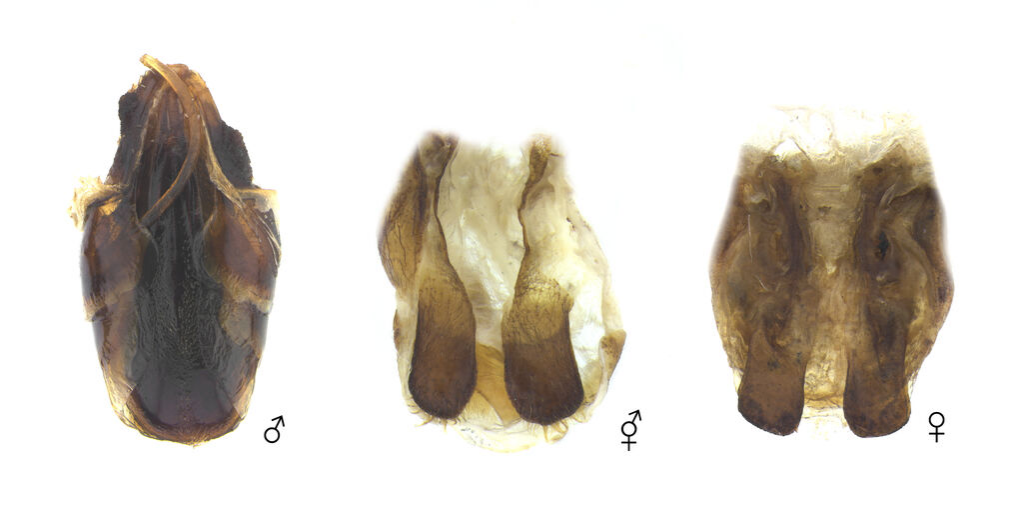
Genitalia of *Dorcustitanuscastanicolor*. From left: male, gynandromorph and female.

The body primarily exhibits female traits (Fig. 6). The head shows asymmetrical dimorphism, with the left side resembling a male and the right side resembling a female (Fig. 7). The surface of the head appears irregular, featuring patchy punctures. Male-type punctures are predominantly located on the left side, while female-type punctures are predominantly located on the right (Fig. 7). The mandibles are asymmetrical, with the left side more developed. The left antenna is slightly larger (Fig. 7). In the central area of the left side of the pronotum, male-type punctures appear in a patchy pattern (Fig. 7). The legs exhibit only female characteristics (short, with a rather wide protibia) (Fig. 6). The genitalia are fully developed and identical to a typical ovipositor (Fig. 8).

#### Notes

In *D.titanuscastanicolor*, the prominent male sexual characteristics include well-developed mandibles, a non-projected canthus, a subrectangular pronotum and relatively long tarsi (Fig. 6). In the observed specimen, most body traits, including the legs and genitalia, exhibited female morphological characteristics. However, irregular patches of both male-type and female-type punctures were noted on the head and pronotum (Figs 6, 7, 8). Hence, this specimen was classified as a gynandromorph. This is the first record for this subspecies.

### Trypoxylus dichotomus septentrionalis

(Kôno, 1931)

52205D92-6483-5CAF-9054-36F051A2459D

#### Materials

- **Type status:** Other material. **Occurrence:** sex: gynandromorph; occurrenceID: BB0266CB-A621-5217-9808-8AFA3591AFA7; **Taxon:** genus: Trypoxylus; specificEpithet: *dichotomusseptentrionalis*; scientificNameAuthorship: (Kôno, 1931); **Location:** country: South Korea; locality: Songyong Lee leg.; **Event:** eventDate: 30.XI.2019 (breed); **Record Level:** basisOfRecord: Preserved Specimen
- **Type status:** Other material. **Occurrence:** sex: 1 male, 1 female; occurrenceID: D3195208-3391-57AF-A0E0-BCAD19B62715; **Taxon:** genus: Trypoxylus; specificEpithet: *dichotomusseptentrionalis*; scientificNameAuthorship: (Kôno, 1931); **Location:** country: South Korea; stateProvince: Jeollanam-do; locality: Is. Jindo, Jukrim-ri, Imhoe-myeon, Jindo-gun; **Event:** eventDate: 4.VIII.2020; **Record Level:** basisOfRecord: Preserved Specimen
- **Type status:** Other material. **Occurrence:** sex: 1 female; occurrenceID: 394BE149-33BB-5433-9E13-1FFA34CF3510; **Taxon:** genus: Trypoxylus; specificEpithet: *dichotomusseptentrionalis*; scientificNameAuthorship: (Kôno, 1931); **Location:** country: South Korea; stateProvince: Jeju-do; locality: Is. Jejudo, Daepo-dong, Seogwipo-si; **Event:** eventDate: 29.VII.2018; **Record Level:** basisOfRecord: Preserved Specimen

#### Description

##### Morphology of the gynandromorph *Trypoxylusdichotomusseptentrionalis*

**Figure 9.**
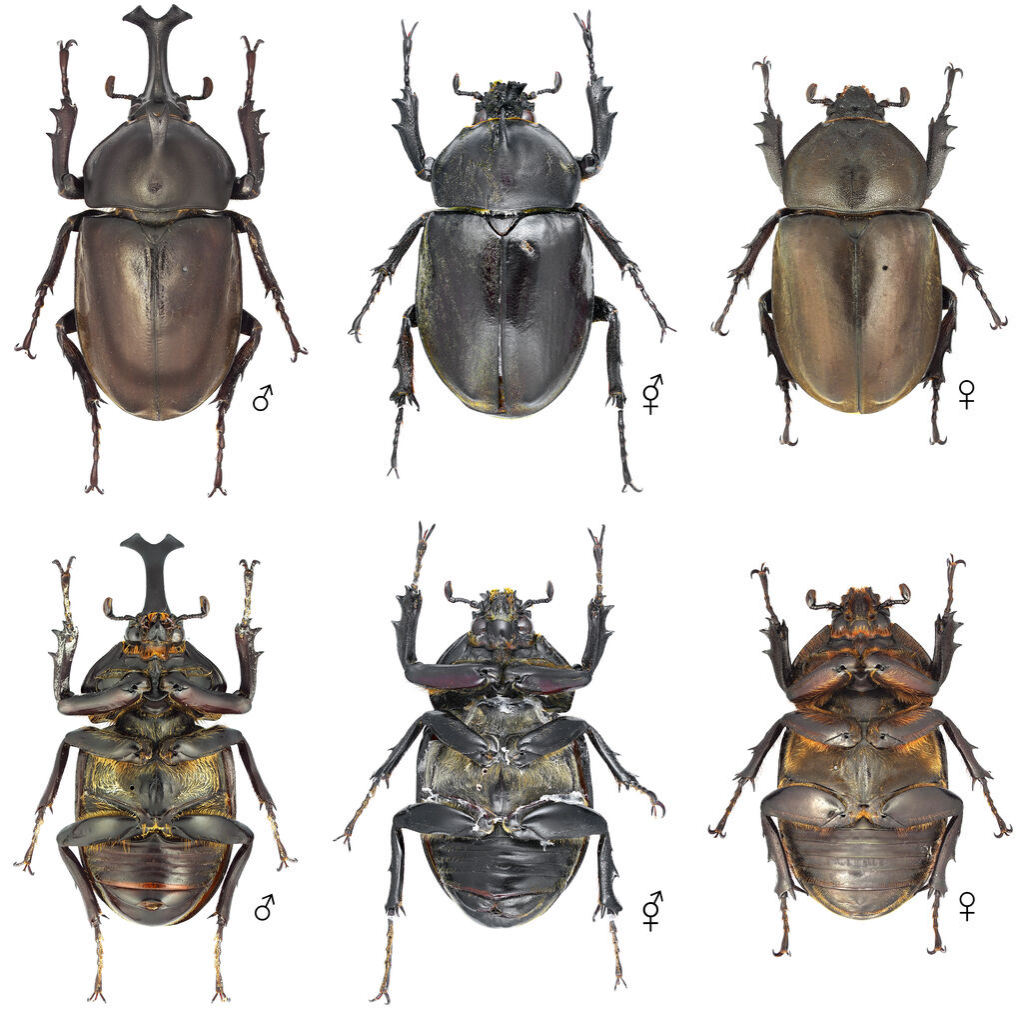
Habitus of *Trypoxylusdichotomusseptentrionalis*, dorsal (above) and ventral (below) views. From left: male, gynandromorph and female.

**Figure 10.**
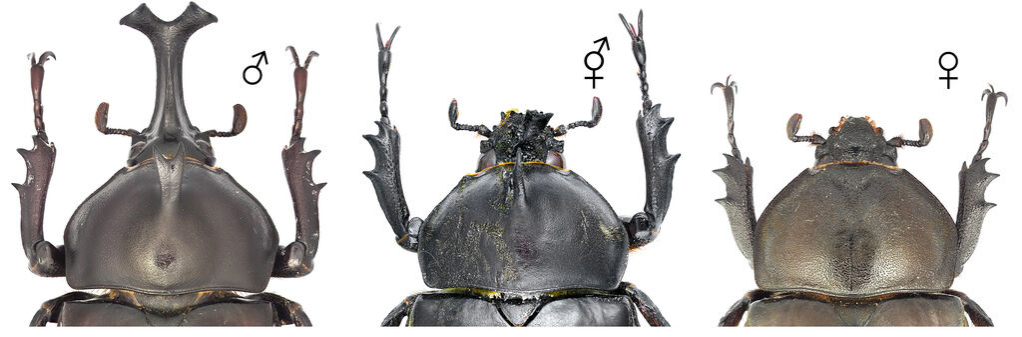
Pronotum and head of *Trypoxylusdichotomusseptentrionalis*. From left: male, gynandromorph and female.

**Figure 11.**
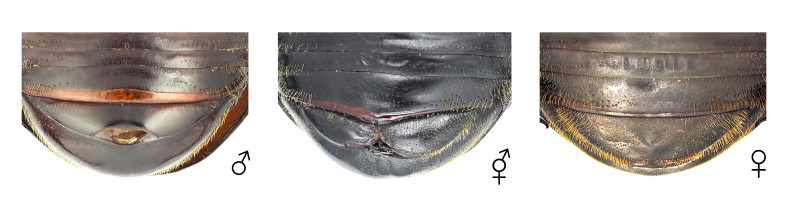
Abdominal sternites of *Trypoxylusdichotomusseptentrionalis*. From left: male, gynandromorph and female.

**Figure 12.**
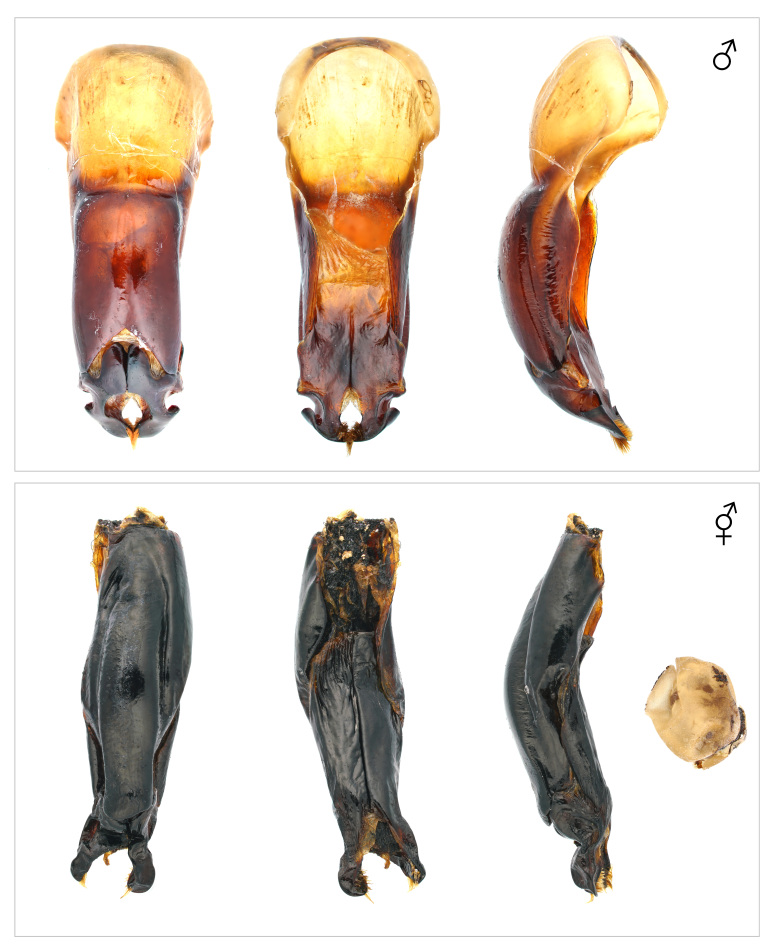
Genitalia and egg of *Trypoxylusdichotomusseptentrionalis*. Above: normal individual; Below: gynandromorph.

The body exhibits a bilateral division of male and female phenotypes, with the left side displaying female characteristics and the right displaying male characteristics (Fig. 9). The head primarily exhibits female traits, but a subrectangular horn is present on the right side of the frons (Fig. 10). The pronotum resembles that of a male, with an incomplete horn on the left side and scattered punctures and pubescence appearing as spots on the surface (Fig. 10). The elytra display female characteristics (dense pale-yellow pubescence) on the left side, while the right side exhibits male characteristics (lacking dense pale-yellow pubescence) (Fig. 9). The legs exhibit a mixture of male and female traits. The protibiae on both sides display female characteristics (rather short and wide, with dense and wide punctures on the surface), but the claws are male-like. The mesotibiae and mesotarsi on both sides display female characteristics (rather short, with two external teeth on the outer margin of the mesotibia). The metatibia and metatarsus are asymmetrical, with the left side displaying female characteristics (a well-developed bidentate apex and a rather short tarsus), while the right side possesses both male (a rather narrow metatibia and a long metatarsus) and female characteristics (two external teeth on the outer margin of the metatibia). The femora overall exhibit male characteristics, except for the left profemur and left metafemur, which display female characteristics (rather wide, with the anterior margin of the profemur covered in dense yellow pubescence) (Fig. 9). The sternites exhibit asymmetry, with a narrow intersegmental space on the left and a broader one on the right (Fig. 11). The genitalia resemble a male aedeagus, but are asymmetrical between the left and right sides, abnormally developed and have paramere apices that are outwardly spread (Fig. 12). Additionally, eight eggs were found alongside the genitalia (Fig. 12).

#### Notes

In *T.dichotomusseptentrionalis*, the primary male sexual characteristics include a well-developed horn on the head and pronotum, a relatively smooth pronotal surface and an elytral surface lacking dense pale-yellow pubescence. In the observed specimen, the body overall displayed a bilateral separation of male and female phenotypes (Figs 9, 10, 11), but the legs exhibited a complex mixture of male and female traits. Based on these male and female morphological distinctions, this specimen was classified as a gynandromorph.

## Analysis

### Updated list and composition of sexual anomaly cases in Coleoptera

**Figure 13.**
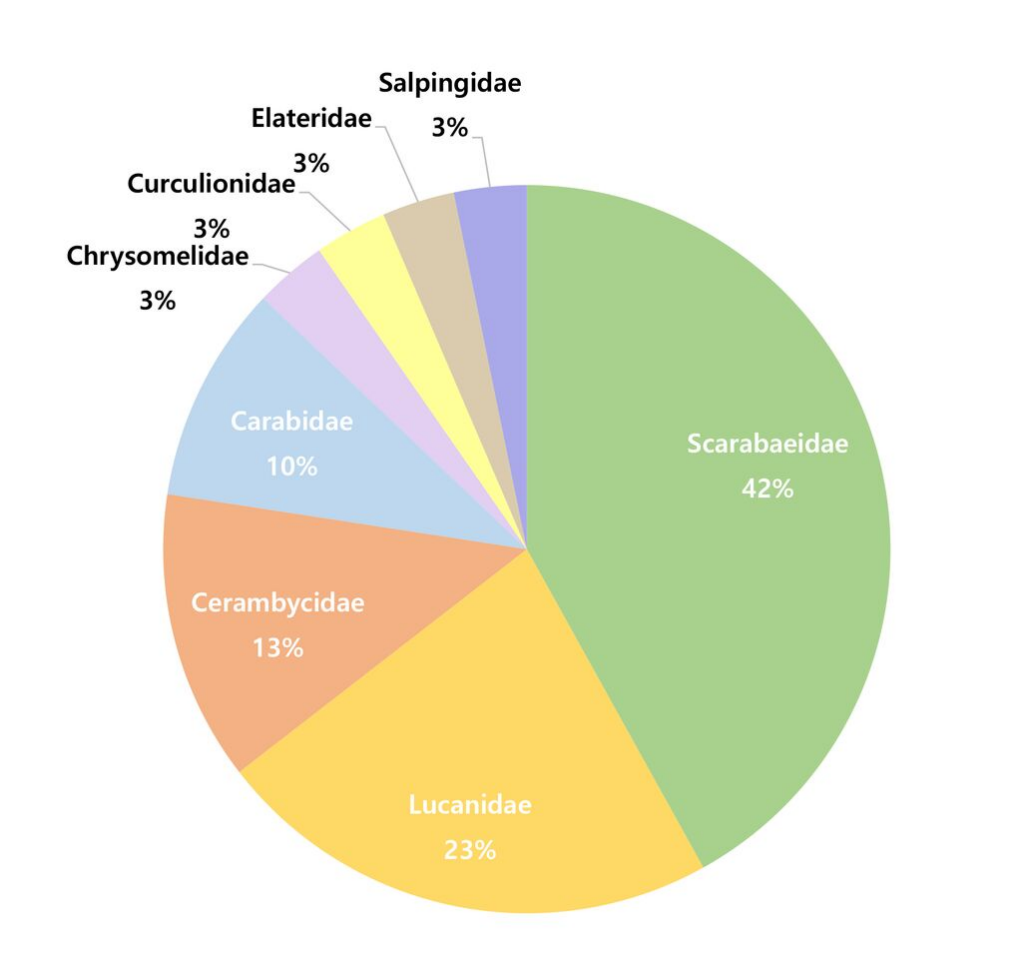
Composition of intersex and gynandromorph cases in Coleoptera.

The compilation of our research findings and updation of intersex and gynandromorphic cases in Coleoptera revealed a total of 31 species across eight families (Table 1). Amongst these, the family Scarabaeidae was found to have the largest proportion of cases (42%), followed by Lucanidae (23%), Cerambycidae (13%) and Carabidae (10%) (Fig. 13).

## Discussion

In arthropods, individuals exhibiting mixed male and female traits have been reported in various insect and non-insect taxa under both natural and experimental conditions (Narita et al. 2010). However, the molecular distinction between gynandromorphs and intersex individuals often remains unclear (Narita et al. 2010). Therefore, in this study, generally accepted definitions were used to differentiate between these conditions.

Amongst the three observed specimens, *P.fortunei* was classified as intersex, as all its body parts, including the genitalia, displayed female characteristics, except for the antennae, which exhibited ambiguous male traits. In contrast, *D.titanuscastanicolor* and *T.dichotomusseptentrionalis* were categorised as gynandromorphs, exhibiting the most common patterns, patchy type and bilateral type, respectively (Fusco and Minelli 2023).

Limited research has been conducted on the mating behaviour of intersex and gynandromorphic insects, though some cases have been reported in certain insect orders, including Ephemeroptera, Orthoptera, Phasmatodea, Hemiptera, Diptera and Hymenoptera (Barth and Bell 1971, Mertins and Coppel 1971, Witherell 1971, Cook 1978, Seow-Choen 1995, Vance 1997, Maeno and Tanaka 2007, Sampson et al. 2010, Matsuo et al. 2018). For example, a gynandromorphic individual of *Bombusignitus* (Smith, 1869) approached a virgin female in a laboratory setting, but took longer than typical males to attempt mating and ultimately failed (Matsuoka 2013). Similarly, a gynandromorphic individual of *Osmiaribiflorisbiedermannii* Michener, 1936 attempted mating, but was unsuccessful (Sampson et al. 2010). Although these individuals may exhibit sex-specific behaviour, they generally fail to achieve successful mating.

Several cases of gynandromorphic or intersex individuals have been reported in beetles (Table 1). However, no study has yet been published regarding their mating behaviour. This study aimed to assess mating behaviour during controlled breeding attempts. Unfortunately, all gynandromorphic individuals perished before observations could be made, limiting the study to *P.fortunei*. When one intersex individual and one typical female were housed together, only aggressive interactions were observed and no mating behaviour was recorded.

Behavioural studies of intersex and gynandromorphic individuals are essential for gaining a deeper understanding of their behaviour. Additionally, such studies can provide valuable insights into their social interactions and reproductive potential under both natural and experimental conditions. Therefore, in addition to reporting intersex and gynandromorphic cases, future research should assess their behavioural interactions with typical males or females.

The compilation of our research findings and list updates identified 31 species across eight families (Table 1). Scarabaeidae accounts for the largest proportion (42%), followed by Lucanidae (23%), Cerambycidae (13%) and Carabidae (10%) (Fig. 13). However, these figures may be limited, as they are based solely on published literature.

One factor contributing to this limitation is that intersex and gynandromorphic individuals, particularly within Coleoptera, often attract collectors due to their rarity and distinctive appearances, leading to high market prices (e.g. on platforms like eBay or specialised insect trading websites). Many such specimens are likely held in private collections, resulting in numerous undocumented cases in scientific literature.

Another factor is that Scarabaeidae and Lucanidae species, which together account for 65% of these cases (Fig. 13), are especially popular as pets (e.g. stag beetles and rhinoceros beetles) (Kawahara 2007). The high demand for these beetles has led to large-scale breeding in commercial facilities and by private breeders, increasing the likelihood of discovering intersex or gynandromorphic individuals compared to other beetle families.

Moreover, in taxa with less pronounced sexual dimorphism, intersex or gynandromorphic individuals may go unnoticed. As a result, it is likely that many undocumented cases exist not only in highly diverse families such as Staphylinidae, Carabidae and Curculionidae, but also in taxa with subtle sexual dimorphism, such as Coccinellidae. Therefore, to improve our understanding of gynandromorphism and intersex phenomena, systematic research and documentation of these taxa are essential.

### Conclusion

This study reviewed and documented 31 beetle species exhibiting intersex or gynandromorphic traits across eight families, including an intersex individual of *P.fortunei* and two gynandromorphic individuals of *D.titanuscastanicolor* and *T.dichotomusseptentrionalis*. Despite the continuous reporting of these cases, significant gaps remain in our understanding of these phenomena, particularly regarding their genetic mechanisms, developmental processes and behavioural implications. To fully comprehend intersex and gynandromorphic occurrences in beetles, an integrated approach combining molecular, genetic and behavioural studies is essential. Future research should focus on behavioural observations to assess their reproductive potential and interactions with typical individuals. Additionally, uncovering the ecological and evolutionary significance of these conditions could provide deeper insights into insect development and sex determination. Expanding systematic research and documentation will be crucial in advancing our knowledge of these rare and fascinating phenomena.

## Supplementary Material

XML Treatment for Pectocera fortunei

XML Treatment for Dorcus titanus castanicolor

XML Treatment for Trypoxylus dichotomus septentrionalis

6869B6F4-B46F-5418-A762-04152A9C9655 10.3897/BDJ.13.e144929.suppl1 Supplementary material 1 Breeding cases and environments used for behavioural observation Data type images Brief description A: breeding case, B: breeding environment setup. File: oo_1241898.jpg https://binary.pensoft.net/file/1241898 Donguk Kim, Sang Eun Hyun, Kwang Shik Choi

## Figures and Tables

**Table 1.** Updated list of intersex and gynandromorph cases in Coleoptera. New records are indicated with an asterisk (*).

**N**	**Family**	**Species**	**References**
1	Carabidae	*Carabusnemoralis*	Hartkorn (1982)
2		Carabus (Leptocarabus) arboreus	Morishita (1991)
3		Pterostichus (Nialoe) musahiensis	Kashara and Karube (1995)
4	Cerambycidae	*Akimerusschaefferi*	Auvray and Auvray (1998)
5		*Ergatesfaber*	Balazuc (1952)
6		*Lepturarubra*	Weber (1913)
7		*Rhagiummordax*	Starzyk (1984)
8	Chrysomelidae	*Cerotomafacialis*	Ruppel (1971)
9	Curculionidae	*Euplatypushintzi*	Beaver (2000)
10	Elateridae*	Pectocerafortunei*	This study
11	Lucanidae	Dorcustitanuscastanicolor*	This study
12		*Dorcustitanuspilifer*	Okushima et al. (2023)
13		*Dorcustitanussakishimanus*	Ogata (1993)
14		*Lucanuselaphus*	Wickham (1903)
15		*Lucanusmaculifemoratus*	Hayashi (1987), Hayashi (1993), Tanikado (1994), Noumi and Kuranishi (2010)
16		*Neolucanusinsularis*	Sakaino and Kawata (1995)
17		*Neolucanusinsulicolainsulicola*	Matsuoka (2013)
18		*Prosopocoilushachijoensis*	Inoue (2013)
19		*Prosopocoilusinclinatusinclinatus*	Hirayama and Shimizu (2012)
20	Salpingidae	*Borosdiscicollis*	Spilman (1953)
21	Scarabaeidae	*Cotinismutabilis*	Deuve (1992)
22		*Dasylepidaishigakiensis*	Tanaka et al. (2006)
23		*Dicranocephaluswallichiiwallichii*	Mizunuma (2002)
24		*Goliathuscacicus*	Ture (2001)
25		*Golofatersander*	Ratcliffe (1989)
26		*Megasomaelephas*	Dechambre (1987)
27		*Megasomaoccidentale*	Blackaller-Bages and Gado-Castillo (1990)
28		*Melolonthajaponica*	Kurosawa (1969)
29		*Paratrichiusdoenitzi*	Kurosawa (1993); Haga (1997)
30		*Polyphyllafullo*	Vasko (2008)
31		*Polyphyllalaticollis*	Kurosawa (1993)
32		*Protaetiaexasperataexasperata*	Shuida (2020)
33		*Trypoxylusdichotomusseptentrionalis*	Kurosawa (1979), Sakurai (2013), This study

## References

[B12384160] Auvray C., Auvray N. (1998). Gynandromorphisme parfait chez le Longicorne *Akimerusschaefferi* Laicharting, 1784 (Col. Cerambycidae).. Entomologie Tourangelle et Ligerienne.

[B12384242] Balazuc Jean (1952). Un *Ergatesfaber* L. gynandromorphe [Col. Cerambycidae]. Bulletin de la Société Entomologique de France.

[B12384251] Barth R. H., Bell William J. (1971). Reproductive Physiology and Behavior of Byrsotria fumigata Gynandromorphs (Orthoptera (Dictyoptera): Blaberidae). Annals of the Entomological Society of America.

[B12384260] Beaver R. (2000). A gynandromorph specimen of *Euplatypushintzi* (Schaufuss) from South Africa (Coleoptera: Platypodidae). Entomologist’s Monthly Magazine.

[B12384269] Blackaller-Bages J., Gado-Castillo L. (1990). A case of gynandromorphy in *Megasomaelephasoccidentalis* Bolivar, Jimenez and Martinez (Coleoptera: Melolonthidae). Coleopterists Bulletin.

[B12384278] Cook R. (1978). The reproductive behavior of gynandromorphic *Drosophilamelanogaster*. Zeitschrift für Naturforschung Section C: Biosciences.

[B12384288] Dechambre R. (1987). Un cas de gynandromorphisme biparti chez *Megasomaelephas* (F.) (Coleoptera, Dynastidae). Annales de la Societe Entomologique de France.

[B12384297] Deuve T. (1992). Origine segmentaire des genitalia ectodermiques mâles et femelles des insectes. Données nouvelles apportées par un gynandromorphe de Coléoptère. Comptes Rendus de l’Académie des Sciences.

[B12384306] Fusco Giuseppe, Minelli Alessandro (2023). Descriptive versus causal morphology: gynandromorphism and intersexuality. Theory in Biosciences.

[B12384324] Haga K. (1997). Gynandromorph individual of *Paratrichiusdoenitzi*. Coleopterists' News.

[B12384315] Hartkorn J. (1982). Ein bemerkenswerter Fund eines Gynanders von *Carabusnemoralis* im sudhessischen Ried Coleoptera: Carabidae.. Entomologische Zeitschrift.

[B12384342] Hayashi N. (1987). Japanese Insects 8 *Lucanusmaculifemoratus*.

[B12384350] Hayashi N. (1993). Morphology and behavior of gynandromorph *Lucanusmaculifemoratus*. Gekkan-Mushi.

[B12384359] Hirayama N., Shimizu T. (2012). Collection of a gynandromorph of *Prosopocoilusinclinatusinclinatus*. Gekkan-Mushi.

[B12384368] Inoue A. (2013). Collection of a gynandromorph of *Prosopocoilushachijoensis*. Gekkan-Mushi.

[B12384377] Kashara S., Karube H. (1995). Occurrence of a gynandromorph of *Pterostichusmusahiensis* [sic] (Carabidae) in Kanagawa Prefecture. Coleopterists' News.

[B12384386] Kawahara Akito Y. (2007). Thirty-foot telescopic nets, bug-collecting video games, and beetle pets: Entomology in modern Japan. American Entomologist.

[B12384395] Kurosawa Y. (1969). Gynandromorph of *Melolonthajaponica*. Coleopterists' News.

[B12384404] Kurosawa Y. (1979). Gynandromorph of *Trypoxylusdichotomus*. Coleopterists' News.

[B12384413] Kurosawa Y. (1993). Abnormality of *Polyphyllalaticollis*. Coleopterists' News.

[B12384422] Lightburn K., van Acker R., Raine N. (2022). The first gynandromorph record of the North American bee *Hylaeusmodestus* (Hymenoptera: Colletidae).. The Journal of the Entomological Society of Ontario.

[B12384469] Maeno K., Tanaka S. (2007). Morphological and behavioural characteristics of a gynandromorph of the desert locust, *Schistocercagregaria*. Physiological Entomology.

[B12384450] Matsuo Koshiro, Kubo Ryohei, Sasaki Tetsuhiko, Ono Masato, Ugajin Atsushi (2018). Scientific note on interrupted sexual behavior to virgin queens and expression of male courtship-related gene fruitless in a gynandromorph of bumblebee, *Bombusignitus*. Apidologie.

[B12384431] Matsuoka S. (2013). Collection of a gynandromorph of *Neolucanusinsulicolainsulicola*. Gekkan-Mushi.

[B12384478] Mertins J. W., Coppel H. C. (1971). bSexual ehavior in gynandromorphs of *Diprionsimilis* (Hymenoptera: Diprionidae). Annals of the Entomological Society of America.

[B12384487] Mizunuma T. (2002). A gynandromorphy of *Dicronocephaluswallichiiwallichii*. Saikaku Tsushin.

[B12384496] Morishita K. (1991). Gynandromorph of Carabus (Leptocarabus) arboreus. Coleopterists' News.

[B12384505] Narita Satoko, Pereira Rodrigo, Kageyama Daisuke, Kjellberg Finn (2010). Gynandromorphs and intersexes: potential to understand the mechanism of sex determination in arthropods. Terrestrial Arthropod Reviews.

[B12384514] Noumi S., Kuranishi R. (2010). Collecting examples of *Lucanusmaculifemoratus* gynandromorph type. Coleopterists' News.

[B12384523] Ogata C. (1993). Special Issue of Tanpo 12. Gekkan-Mushi.

[B12384532] Okushima Y., Kawate H., Kawate Y. (2023). Record of a gynandromorph in *Dorcustitanuspilifer*. Gekkan-Mushi.

[B12384541] Ratcliffe B. (1989). A case of gynandromorphy in *Golofatersander* Burmeister (Coleoptera: Scarabaeidae). Coleopterists Bulletin.

[B12384550] Ruppel R. (1971). An asymmetrical gynandromorph of *Cerotomafacialis* (Coleoptera: Galerucidae). Great Lakes Entomologist (Michigan Entomological Society).

[B12384577] Sakaino H., Kawata K. (1995). A record of *Neolucanusinsularis* gynandromorph type. Coleopterists' News.

[B12384586] Sakurai T. (2013). Eclosion of a gynandromorph in *Trypoxylusdichotomusseptentrionalis*. Gekkan-Mushi.

[B12384595] Sampson B. J., Kirker G. T., Werle C. T. (2010). Morphology, courtship and mating of a mixed bilateral gynander of *Osmiaribiflorisbiedermannii* Michener (Hymenoptera: Megachilidae). Journal of the Kansas Entomological Society.

[B12384613] Seow-Choen F. (1995). Two more gynandromorphs of the Malayan jungle nymph, *Heteropteryxdilatata* (Phasmida) with notes on captive behaviour. Bulletin of the Amateur Entomologists’ Society.

[B12384640] Shuida S. (2020). Collection of a Gynandromorph of *Protaetiaexasperataexasperata* in Amami Oshima. Gekkan-Mushi.

[B12384622] Spilman T. J. (1953). An odd case of gynandromorphism in the external genitalia of *Borosdiscicollis* (Salpingidae). The Coleopterists' Bulletin.

[B12384631] Starzyk J. (1984). Rare case of bilateral gynandromorphism in *Rhagiummordax* (Deg.) (Col., Cerambycidae). Entomologist’s Monthly Magazine.

[B12384649] Tanaka Seiji, Yukuhiro Fumiko, Wakamura Sadao (2006). Sexual dimorphism in body dimensions and antennal sensilla in the white grub beetle, *Dasylepidaishigakiensis* (Coleoptera: Scarabaeidae). Applied Entomology and Zoology.

[B12384658] Tanikado M. (1994). Gynandromorph *Lucanusmaculifemoratus* Collected in Sekinomiya Town. Iratsume.

[B12384667] Ture A. (2001). Cas de gynandromorphisme chez *Goliathuscacicus* en Cote d’Ivoire. Lambillionea.

[B12384676] Vance SA. (1997). Morphological and behavioural sex reversal in mermithid-infected mayflies. Proceedings of the Royal Society of London. Series B: Biological Sciences.

[B12384686] Vasko B. (2008). Occurencies of teratosus [teratosis] and gynandromorphism among some species of beetles of the genus *Polyphylla* (Coleoptera, Melolonthidae). Vestnik Zoologii.

[B12384695] Weber L. (1913). Ein gynandromorphes Exemplar von *Lepturarubra* L. Entomologische Blätter Berlin.

[B12384713] Wickham H. (1903). Gynandromorphism in *Lucanuselaphus*. Canadian Entomologist.

[B12384704] Witherell Peter C. (1971). Note on behavior of gynandroniorphic honey bees, *Apismellifera*. Annals of the Entomological Society of America.

