# Supplementary figures and images for: First report of an intersex individual of the click beetle Pectocerafortunei (Elateridae) and additional cases of gynandromorphism in Coleoptera (Lucanidae, Scarabaeidae)
Source: Biodivers Data J. 2025 Feb 20;13:e144929. doi: 10.3897/BDJ.13.e144929 (PMC11868802; doi:10.3897/BDJ.13.e144929)

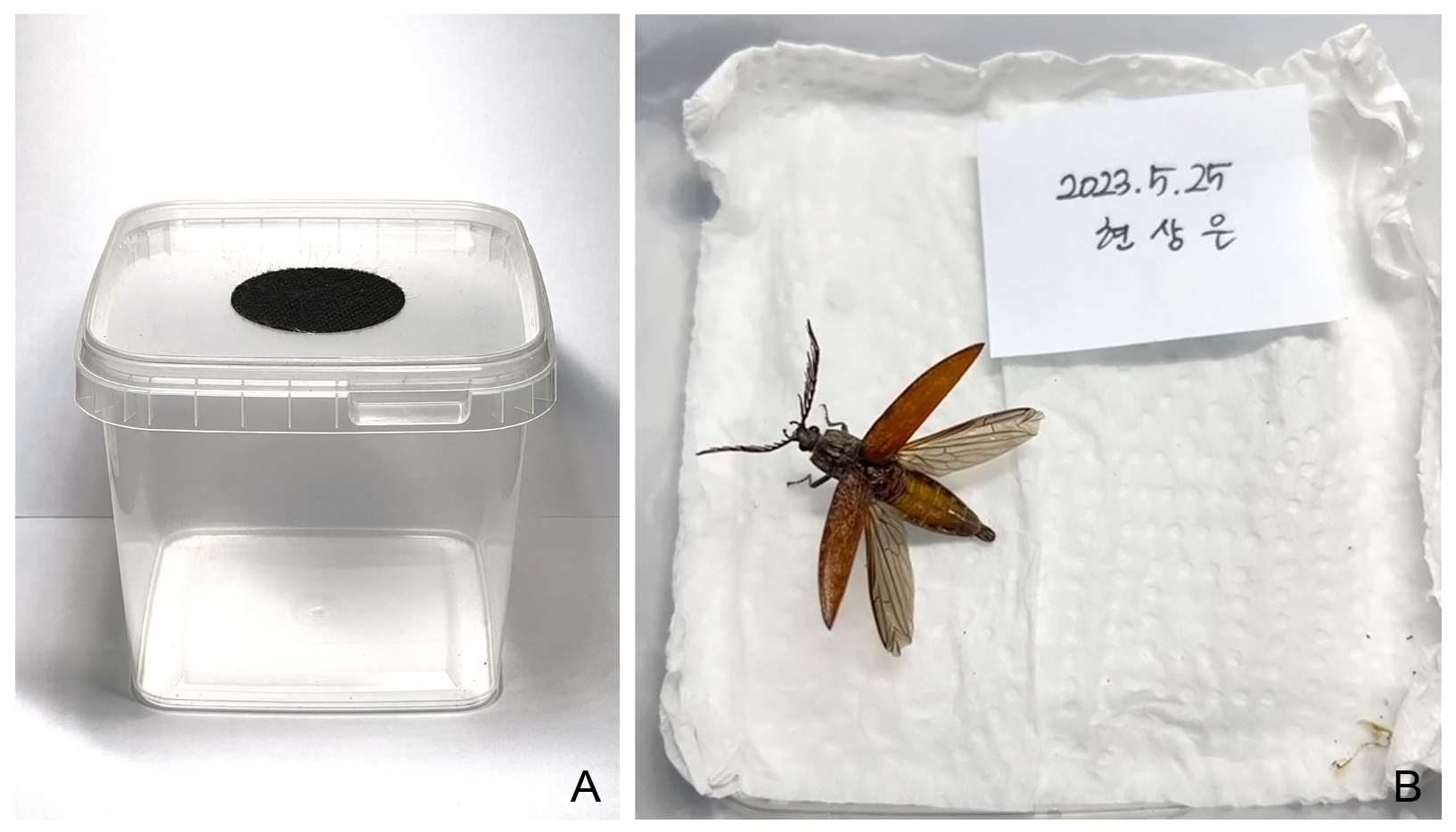

Supplement: Supplementary material 1 — Breeding cases and environments used for behavioural observation [file bdj-13-e144929-s001.jpg]
